# Predictors of Functional Outcome in Unstable Ankle Fractures Treated Surgically – A Prospective Cohort Study

**DOI:** 10.5704/MOJ.2103.013

**Published:** 2021-03

**Authors:** G Balaji, S Bhukya, S Nema, M Rajeswari, V Vellaipandi

**Affiliations:** 1 Department of Orthopaedics Surgery, Jawaharlal Institute of Postgraduate Medical Education and Research (JIPMER), Pondicherry, India; 2 Department of Biostatistics, Jawaharlal Institute of Postgraduate Medical Education and Research (JIPMER), Pondicherry, India; 3 Department of Orthopaedics, Mahatma Gandhi Medical College and Research Institute, Pondicherry, India

**Keywords:** ankle, fracture, outcomes, trauma

## Abstract

**Introduction:**

Unstable ankle injuries require anatomical reduction and stabilisation for optimal outcome. In spite of adequate care, a few patients have poor outcome. In this study, we assessed the risk factors that predict the clinical outcomes in surgically treated unstable ankle fractures.

**Material and methods:**

This prospective cohort study was conducted on 68 patients who underwent surgical management for an unstable ankle injury. Demographic details, fracture type and associated medical comorbidities were recorded. Pre-operative radiographic assessment was done for all patients. At the end of one year follow-up, clinical (American Orthopaedic foot and ankle society-AOFAS and Olerud-Molander ankle - OMAS) scores and radiological parameters were assessed and analysed.

**Results:**

Fracture dislocation (0.008), diabetes mellitus (0.017), level of alchohol consumption (0.008) and pre-operative talocrural angle (TCA) > 100° (0.03) were significant predictors of poor outcomes as per AOFAS. Fracture dislocation (0.029), diabetes mellitus (0.004), pre-operative TCA > 100° (0.009), female gender (0.001), age more than 60 years (0.002) and open injuries (0.034) had significantly poor outcome as per OMAS. Other parameters (smoking, hypertension, classification, syndesmotic injury, medial clear space and tibiofibular overlap) did not affect the outcome significantly.

**Conclusion:**

Our study showed that poor outcome predictors in unstable ankle fractures are age >60 years, female gender, diabetes mellitus, alcohol consumption, fracture dislocation, open fractures and pre-op TCA >100°.

## Introduction

The ankle, a complex hinge joint, is responsible for transmitting the whole body weight to the ground. Injury to this joint results in significant morbidity due to alteration in the joint biomechanics. The management of ankle fractures is a challenging task for any surgeon due to a wide variety of fracture patterns and minimal soft tissue coverage. Fracture dislocation and displaced malleolar fractures contribute to ankle instability. These unstable fractures should be anatomically reduced and stabilised to enable early mobilisation. The aim of surgery is to provide a pain-free stable ankle.

Despite a high incidence of ankle fractures, only few studies have been conducted to assess outcomes^1-7^. Outcomes of ankle fractures after treatment have been investigated using various functional scores. Almost two third of the patients achieve good to excellent results based on the scores, but outcome in the remaining one third have been reported as acceptable or poor. They have residual pain, loss of ankle motion and osteoarthritis^7-10^. Hence, it is important to identify the factors that can influence the outcome and thereby help clinicians to predict the prognosis.

Multiple factors related to patients, surgeon, type of injury and fixation techniques govern the outcome of these injuries. Very few studies have reported factors that affect outcome in ankle fractures^2,4,5,6,11^. Most of these studies concentrated on demographic and clinical variables and compared them to radiologic parameters. Hence in this study, we assessed the functional outcomes and the risk factors including pre and post-operative radiologic parameters that predict clinical outcomes in surgically treated unstable ankle fractures.

## Material and Method

This prospective cohort study was conducted from 1st January 2016 to 31st December 2018 at an apex trauma centre for treatment of fractures. The study was approved by the Institute’s review board. Skeletally mature patients with unstable ankle fractures within seven days from the time of injury were included in this study. Displaced medial malleolar fractures > 2mm, bimalleolar, trimalleolar fractures and fracture dislocation were considered as unstable fractures. Isolated lateral malleolar fractures with associated medial clear space widening of > 4mm or presence of syndesmotic widening was also considered as unstable injury. Polytrauma patients, those with associated bony injuries of ipsilateral limb, chronic inflammatory arthropathy, chronic peripheral neurological disorders, pathological fractures, previous history of trauma/infection, associated physical or mental conditions that will affect the compliance with the study were excluded.

**Fig. 1:**
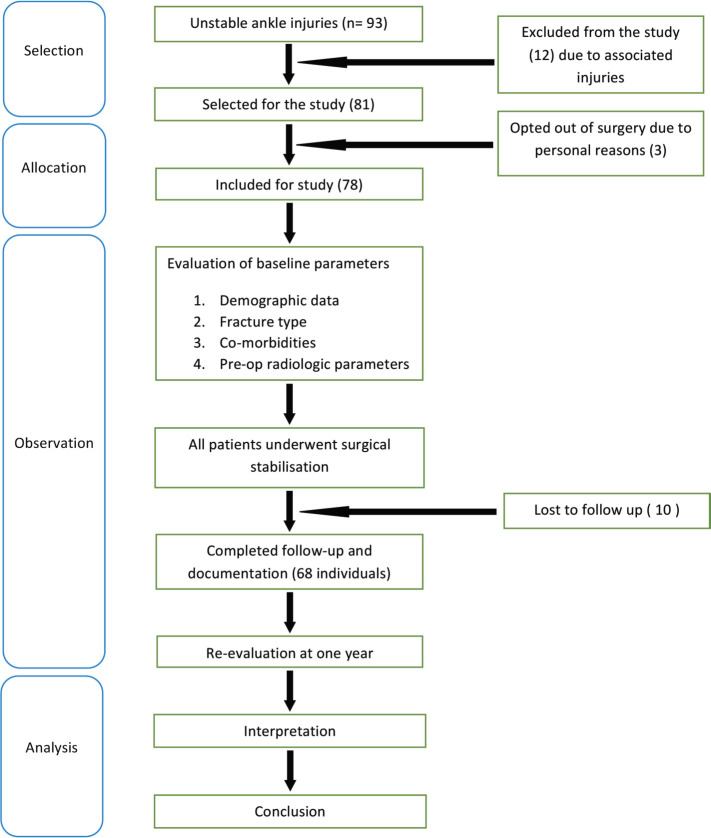
Flowchart showing study methodology.

Once patients have been selected for surgery, their demographics, fracture type and associated medical comorbidities were recorded. Pre-operative radiological assessment was done by measuring the medial clear space (MCS), talocrural angle (TCA), and tibiofibular overlap (TFO) in mortise view. The measurements were done using our hospital picture archiving and communication system (PACS) [General Electric Healthcare Centricity version 12 Chicago, Illinois, United States]. In trauma patients, it is difficult to obtain a mortise view because of pain. Therefore, the radiograph beam was angled 15-20° laterally to achieve the view. Computer tomography (CT) scan with 3D reconstruction was done for all trimalleolar fractures to assess the fracture pattern, fracture comminution and size of the posterior malleolus fragment. Surgical stabilisation was performed by open reduction and internal fixation of the lateral /medial malleoli. Fixation of the posterior malleolus was done directly or indirectly if the fragments remained displaced or if they comprised more than one-third of the joint surface on the lateral radiograph/CT scan. Intra-operative ankle stability was tested by stress radiography to look for syndesmotic injury. If the stress test was positive, syndesmosis was stabilised. All patients were immobilised for four to six weeks with a below knee splint. All patients with closed injuries received one dose of parenteral antibiotic pre-operatively and one dose six hours post-surgery. Antibiotics were continued for five days in those with open injuries. Patients were allowed to gradually weight-bear from eight weeks post-surgery.. Weight bearing was delayed for another two to four weeks in patients older than 50 years of age, diabetics and in those with poor compliance. At the end of one year, patients were assessed clinically (American Orthopaedic foot and ankle society-AOFAS and Olerud-Molander ankle - OMAS score) and radiologically (MCS, TCA, TFO). Study methodology is shown in Fig. 1.

Statistical analysis was done using statistical package for social sciences [IBM Corp. Released 2010. IBM SPSS Statistics for Windows, Version 19.0. Armonk, NY: IBM Corp.]. Association of AOFAS and OMAS scores with demographic and radiological variables were measured using Fisher’s exact test. P-value <0.05 was considered significant. The data collected were analysed and correlated to determine the predictors of functional recovery.

## Results

A total of 78 patients were recruited into the study. Ten patients were lost to follow-up accounting for 68 patients at the time of final evaluation (one year). The mean age of the patients was 39.9 ± 13.15. There were 55(79%) males and 13(21%) females. There were 31(45%) patients below 40 years, 33(48%) between 40-60 years and 4(5.8%) above 60 years in the group studied. Fifteen (23%) patients had open ankle fractures while 53(77%) had closed fractures. There were 4(5.8%) fracture dislocations.

The most common injury reported was bimalleolar fracture in 38 (55%) patients. Isolated medial, lateral malleolar and trimalleolar fractures accounted for 14(20%), 7(10%) and 9(11%) patients, respectively. The most common fracture type reported was supination-external rotation 24(35%), followed by pronation-abduction 19(28%), supination-adduction 14(21%) and pronation-external rotation 11(16%) as per Laugh- Hansen classification system. There were 40(59%) Weber-B, followed by Weber-A 15(27%) and Weber-C 13(24%) cases. There were 13 patients (19%) with diabetes mellitus, 12 patients (17%) had a history of chronic alcohol intake, 6 patients (8%) had hypertension and 8 patients (11%) were smokers in the group studied.

**Fig. 2:**
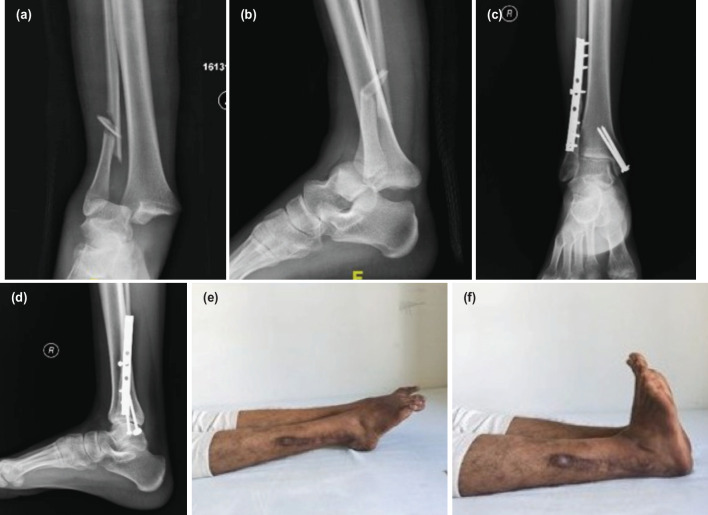
(a, b) Pre-operative radiograph of a 20-year-old male showing ankle fracture dislocation and (c, d) the follow-up radiograph at the end of one year. (e, f) Shows the clinical ankle range of motion at the final follow-up.

According to AOFAS score, 35%, 40%, 15% and 10% had excellent, good, fair and poor outcomes respectively at the end of one year. Based on OMAS, 26% excellent, 41% good, 23% fair and 17% poor outcomes were recorded (Fig. 2).

Mean time to union was 10 weeks (8 weeks to 13 weeks). Mean pre-op MCS was 7mm (0 to 12mm). A 25 (36%) had < 4mm and 43 (63%) had >4mm MCS pre-operatively. At the end of one year, mean MCS was 2mm (2mm to 6mm) with < 4mm group 52 (76%) patients and > 4mm group had 15(22%) patients. Pre-op mean of TCA was 97° (69 to 118). A 30(44%) had <90°, 22(32%) had 90-100° and 15(22%) had > 100°, whereas the post op TCA mean was 81° (74 to 92) with <90 group of 14(20%), 90 to 100 group of 53 (77%) and >100 group had 2(3%) patients. With regards to pre-op TFO, 48 patients had TFO < 1mm while 20 had > 1 mm, 50% had TFO < 1mm post-operatively.

**Fig. 3:**
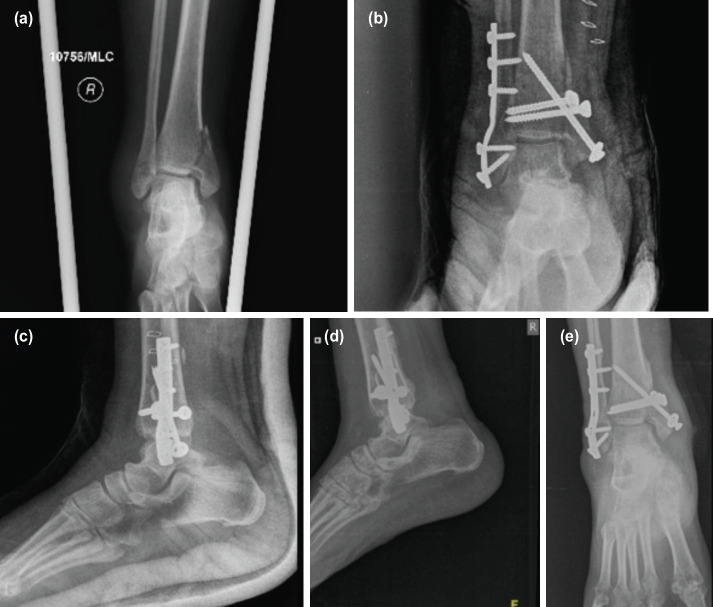
(a) Pre-operative radiograph of a 41-year-old female with bimalleolar ankle fracture who was operated. (b, c) Shows the immediate post-operative radiograph and (d, e) shows medial malleolus non-union at the end of one year follow-up.

**Fig. 4:**
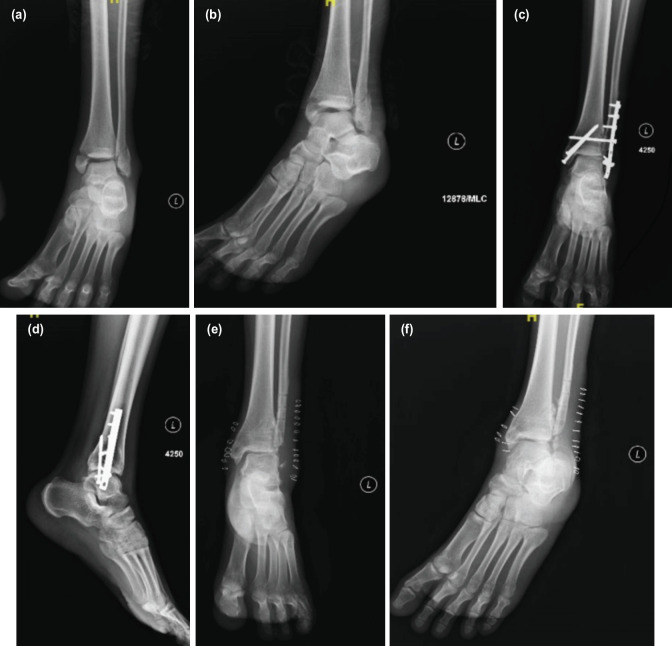
(a, b) Pre-operative radiograph of a 22-year-old male with closed bimalleolar ankle fracture who was operated. (c, d) Shows immediate post-operative radiographs. (e, f) Shows destruction of distal end of lateral malleolus secondary to infection for which implant removal was done.

Complications were recorded in 18% (12) patients. Seven patients had superficial infection which healed with regular dressings. Two patients had deep infection which required hardware removal after union. One patient each had infected non-union of the lateral malleolus, non-union medial malleolus and malunited medial malleolus (Fig. 3,4). Twenty patients (29%) in our study had moderate to severe pain as per AOFAS which is less when compared to other studies. A total of 12(18%) of our patients had ankle stiffness.

The influence of various variables on the clinical outcomes (AOFAS and OMAS) is shown in Tables I-IV .

**Table I:** Table showing the variables that affect the AOFAS score

Variables	AOFAS				P-Value
	Poor	Fair	Good	Excellent	
Sex					
Male	2	13	16	24	0.33
Female	0	5	6	2	
Age					
< 40 Yrs.	1	5	7	18	0.196
40 - 60 Yrs.	1	11	13	8	
> 60 Yrs.	0	2	2	0	
Type Of Fracture					
Open	2	6	3	4	0.119
Closed	0	12	19	22	
Fracture Dislocation					
Yes	0	4	0	0	0.008
No	2	14	22	26	
Diabetes					
Yes	0	7	5	1	0.017
No	2	11	17	25	
Hypertension					
Yes	2	17	19	24	1
No	0	1	3	2	
Alcohol					
Yes	2	4	1	5	0.008
No	0	14	21	21	
Smoking					
Yes	0	2	3	3	1
No	2	16	19	23	
Lauge Hansen Classification					
Sadd	0	4	4	6	0.419
Ser	2	6	6	10	
Pabd	0	7	8	4	
Per	0	1	4	6	
Weber Classification					
Type A	2	2	5	6	0.173
Type B	0	11	13	16	
Type C	0	5	4	4	
Syndesmotic Injury					
Yes	9	12	15	12	0.173
No	3	5	9	11	
Syndesmotic Injury Group					
Operated	1	0	5	7	0.03
Non-Operated	8	10	14	3	

**Table II:** Table showing variables that affect the OMAS score

Variables	OMAS				P-Value
	Poor	Fair	Good	Excellent	
Sex					
Male	2	14	10	29	0.001
Female	1	3	8	1	
Age					
< 40 Yrs.	1	6	3	21	0.002
40 - 60 Yrs.	1	11	12	9	
> 60 Yrs.	1	0	3	0	
Type Of Fracture					
Open	1	7	3	4	0.034
Closed	2	10	15	26	
Fracture Dislocation					
Yes	1	2	1	0	0.029
No	2	15	17	30	
Diabetes					
Yes	2	4	6	1	0.004
No	1	13	12	29	
Hypertension					
Yes	1	0	4	1	1
No	2	17	14	29	
Alcohol					
Yes	2	4	1	5	0.05
No	1	13	17	25	
Smoking					
Yes	0	2	2	4	1
No	3	15	16	26	
Lauge Hansen Classification Sadd	0	3	3	8	0.815
Ser	2	6	8	8	
Pabd	1	6	4	8	
Per	0	2	3	6	
Weber Classification					
Type A	1	1	5	8	0.512
Type B	2	11	9	18	
Type C	0	5	4	4	
Syndesmotic Injury					
Yes	11	10	14	13	0.227
No	4	6	10	8	
Syndesmotic Injury					
Operated	1	3	2	7	0.002
Non-Operated	7	9	12	7	

**Table III:** Table showing the various radiological parameters that influence the AOFAS score

Radiological Variables	AOFAS					P-value
Pre-op TCA	<90	1	10	9	14	0.03
	90-100	1	5	9	9	
	>100	2	2	3	3	
TCA at six months	<90	2	1	2	7	0.22
	90-100	0	17	19	18	
	>100	0	0	1	1	
Pre-op MCS	<4 MM	0	6	7	12	0.516
	>4 MM	1	6	9	7	
MCA at six months	<4 MM	1	13	20	27	0.725
	>4 MM	1	3	2	3	
Pre-op TFO	<1 MM	12	11	13	12	0.420
	>1MM	4	6	10	8	
TFO at six months	<1 MM	3	13	7	11	0.216
	>1MM	1	1	12	10	

**Table IV:** Table showing the various radiological parameters that affect the OMAS score

Radiological Variables	OMAS					P-value
Pre-op TCA	<90	0	7	6	15	0.009
	90-100	1	5	6	12	
	>100	1	5	2	5	
TCA at six months	<90	1	3	2	6	0.085
	90-100	2	14	15	23	
	>100	0	0	1	1	
Pre-op MCS	<4 MM	1	6	7	13	0.817
	>4 MM	1	7	4	9	
MCA at six months	<4 MM	2	13	15	26	0.725
	>4 MM	1	2	3	4	
Pre-op TFO	<1 MM	10	12	13	12	0.420
	>1MM	4	6	10	8	
TFO at six months	<1 MM	11	10	14	13	0.216
	>1MM	4	6	10	8	

Fracture dislocation (0.008), diabetes mellitus (0.017), alcoholics (0.008) and pre-operative TCA > 100° (0.03) were significant predictors of poor outcomes as per AOFAS. Fracture dislocation (0.029), diabetes mellitus (0.004), preoperative TCA > 100° (0.009), female gender (0.001), age more than 60 years (0.002) and open injuries (0.034) had significantly poor outcome as per OMAS. Other parameters (smoking, hypertension, classification, syndesmotic injury, medial clear space and tibiofibular overlap) did not affect the outcome significantly.

## Discussion

Unstable ankle fractures are usually treated surgically in an effort to achieve a painless stable ankle joint. Though most of patients show good to excellent outcomes postsurgery, some patients have residual pain, loss of ROM (range of motion) and progression to arthritis of the ankle joint. In this study, we have assessed the risk factors which influence the short term functional outcomes of ankle fractures. Ten patients were lost to follow-up in our study as they came from distant places and probably could not afford to travel for every visit due to financial constraints.

Out of 68 patients in our study, 35%, 45%, 19% and 10% had excellent, good, fair, and poor outcomes based on AOFAS score. Understandably males, non-alcoholics, younger patients with closed injuries without associated dislocation and those with no comorbidities had excellent and good outcomes in our study. Radiologically, MCS of less than 4mm was associated with excellent and good outcomes, though it failed to reach statistical significance. The findings of this study are similar to earlier studies^1-7^.

Presence of Syndesmotic injury, pre-operative tibiofibular overlap of less than 1mm and pre-operative TCA 90-100° on radiographs were predictors of poorer outcomes in our study, though these factors failed to reach statistical significance. Pre-operative talocrural angle > 100° was a significant predictor of poor outcome in our study. Failure of syndesmotic stabilisation resulted in poor outcomes which was statistically significant. Kohake *et al*^12^ conducted a study in a total of 21 syndesmotic rupture patients with a mean follow-up of 6.6 years and found that syndesmotic rupture does not affect clinical and radiological outcome parameters, but it does lead to a significant restriction in dorsiflexion of the ankle joint.

Investigators have reported poorer outcomes of ankle injuries in diabetics and alcoholics. Post-operative hyperglycaemia, prolonged hospital stay, osteoporotic bones, low immunity and delayed wound healing with high risk of infection have been attributed to poorer outcomes in these patients.

A total of 20(29%) patients in our study had moderate to severe pain as per AOFAS and 12(18%) of our patients had ankle stiffness which is less compared to other studies. Nilsson *et al*^13^ reported persistence of pain, stiffness, and swelling reduced ADL in 60% at 1 year in elderly patients. Hong *et al*^14^ reported that with tri-malleolar fractures 52 to 54% had ankle pain, 61 to 69% had ankle stiffness and 47% had ankle swelling at 1 year follow-up. This finding could be because in our study group elderly patients were less accounting for only 5.8% and also trimalleolar fractures were only 11%. Also, evolution of better methods of fixation, low profile implants in the current scenario may further reduce the complications in future.

Strength of the study was that it was prospective in nature with high follow-up rates (87%) up to one year. The study had a predefined radiological and functional outcome measure. We addressed syndesmotic injuries during surgery and found that patients who underwent syndesmotic stabilisation had excellent to good outcomes in that subgroup.

The short duration of follow-up and failure to compare different modes of fixation were some of the limitations of this study. The cases were operated by multiple surgeons at all levels and that also would have caused bias in outcomes. A long term follow-up would have given a better idea about arthritis.

## Conclusion

To conclude, surgical management is the mainstay of treatment in unstable ankle fractures and the poor outcome predictors are age > 60 years, female gender, diabetes mellitus, excessive alcohol intake, fracture dislocation, open fractures and pre-op TCA > 100°.
